# Challenges in managing neonatal ileal atresia in limited-resource settings: a case report

**DOI:** 10.11604/pamj.2024.48.125.44315

**Published:** 2024-07-23

**Authors:** Frank Martin Sudai, Macrice Yakayashi, Lusajo Mwagobele, Peter Kafubhi, Habakuki Ntabudyo, Kasili Joseph Magembe, Lazaro Yohana Madeha, Stanley Zakaria Binagi, Joseph Nangawe, Jesca Paul Lebba

**Affiliations:** 1 Ministry of Health, Maweni Regional Referral Hospital, Kigoma, Tanzania,; 2 President´s Office, Region Authorities and Local Governments, Region Health Management Team, Kigoma, Tanzania

**Keywords:** Ileal atresia, neonatal intestinal obstruction, cecostomy, case report

## Abstract

Neonatal ileal atresia is a form of intestinal obstruction characterized by narrowing, complete closure, or absence of a segment of the ileum. This case involves a 5-day-old female neonate presented with abdominal distension and bilious vomiting. The neonate, delivered via spontaneous vertex birth weighing 2.9 kg, showed no fever and maintained stable vital signs during examination. Physical assessment revealed abdominal distension, hyper-tympanic areas, dullness in the lower quadrants, reduced bowel sounds, and a patent anus with an empty rectum. Imaging studies confirmed intestinal obstruction from dilated small bowels, leading to explorative laparotomy identifying ileal atresia, necessitating cecostomy placement. Despite surgical intervention, the neonate experienced nutritional complications and unfortunately passed away three days postoperatively. This case underscores the complexities of early diagnosis and management in neonates with intrauterine conditions, particularly in resource-limited settings with limited access to total parenteral nutrition.

## Introduction

Intestinal atresia is a congenital condition characterized by a complete blockage of the intestinal lumen, commonly resulting in bowel obstruction in newborns [1]. This condition can occur at any point along the gastrointestinal tract. Jejuno-ileal atresia (JIA) is the predominant form observed in pediatric patients among the different types of intestinal atresia [2]. Jejuno-ileal atresia typically originates from a vascular event during fetal development that impacts the mesenteric vessels in the midgut, leading to ischemic necrosis and the formation of a blind proximal loop with a distal atretic segment [1].

## Patient and observation

**Patient information:** we report a case of a 5-day-old female baby born by spontaneous vertex delivery weighing 2.9 kg at gestational age 36 weeks and 6 days. The mother reported that the child had experienced episodes of greenish projectile vomitus; no yellowish discoloration of eyes or skin, and no history of fever. The mother booked at 16 weeks and attended two antenatal care (ANC) visits; no ultrasound was done during all visits.

**Clinical findings:** alert, afebrile, not dyspneic, dehydrated with stable vitals (SPO_2_ 99% in room air, PR=160 bp, RR 53 bpm, T 36.9, weight 2.2 kg, length 50 cm) per abdomen mild abdominal distension symmetrically, some areas of tympanic and dullness in lower quadrant, reduced bowel sounds and patent anus with empty rectum.

**Timeline of current episode:** admitted on 13^th^ April 2024, underwent explorative laparotomy on 17^th^ April 2024 and the patient succumbed death on 20^th^ April 2024.

**Figure 1:**
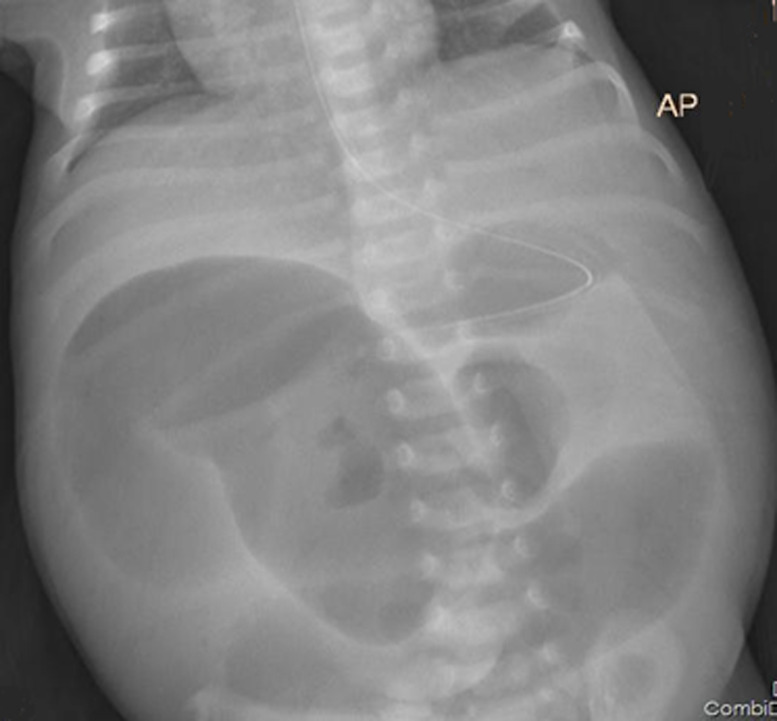
supine pre-operative X-ray which shows dilated small bowel

**Diagnostic assessment:** laboratory investigations included; CBC (had normal leukocyte count 10.9, hemoglobin 20.9, platelets 370) electrolytes (sodium 131, potassium 6.2, and chloride 96), blood group A positive, serum creatinine 30.7, random blood glucose 4 mmoL/L imaging studies ultrasound showed dilated large bowels with massive echogenic fluid collection, supine abdominal X-ray revealed dilated bowels (Figure 1).

**Diagnosis:** intestinal obstruction secondary to congenital GI malformation was highly suspected, other diagnoses were moderate hyperkalemia and mild hyponatremia. After explorative laparotomy, ileal atresia type II was confirmed.

**Figure 2:**
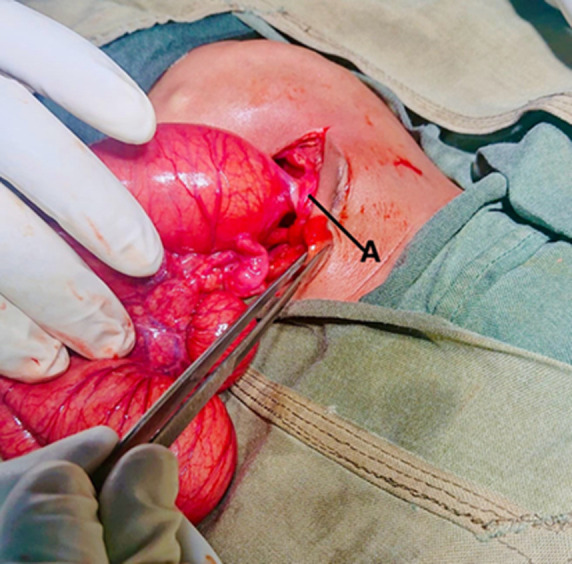
attachment of cecum at the umbilicus

**Figure 3:**
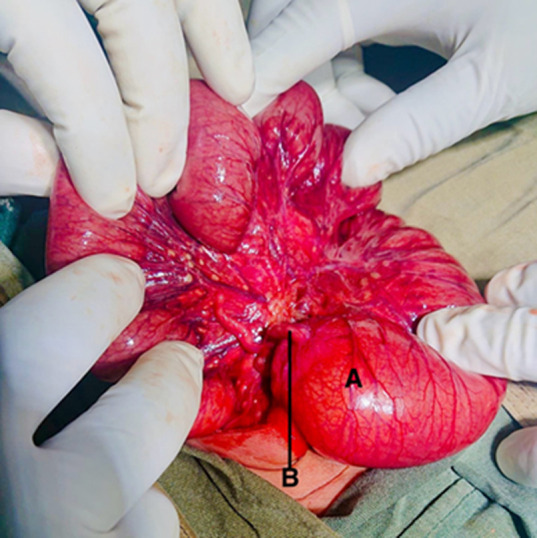
A) distended cecum with terminal ileum; B) appendix

**Figure 4:**
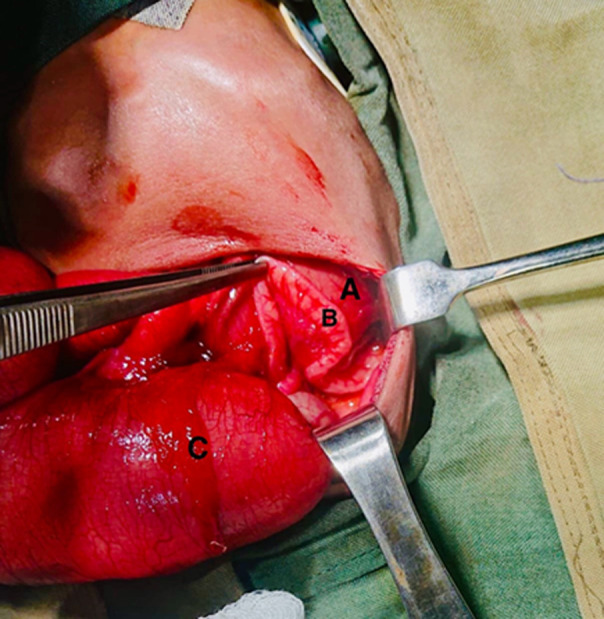
A) distended stomach; B) collapse of the transverse colon; C) distended cecum and terminal ileum

**Figure 5:**
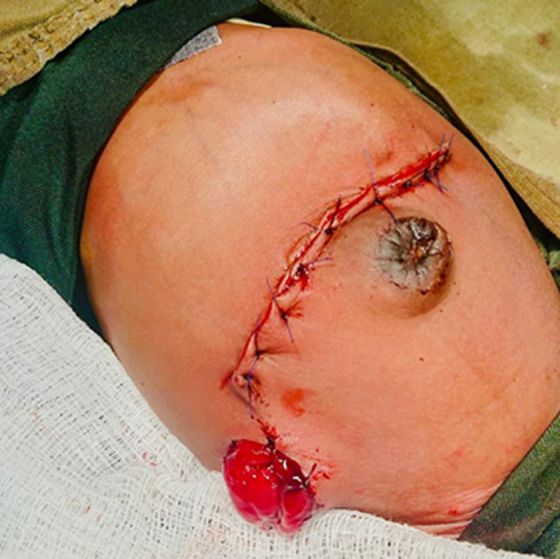
post cecostomy

**Therapeutic interventions:** during admission, the patient was kept nil per oral, and a nasogastric tube was inserted for decompression. The patient received Ampiclillin + Cloxacillin IV 110 mg 8 hours, Gentamycin 15 mg OD, and neonatal fluid (consisting of dextrose 10%, normal saline, and Ringer’s lactate) for hydration and electrolyte balance correction. Once electrolytes were normalized (potassium 5.3 and sodium 135), the patient underwent an explorative laparotomy on the third-day post-admission. Intraoperatively, attachment of caecum to the umbilicus (Figure 2) was distension from the stomach to the cecum (Figure 3), with no connectivity to the ascending colon. Total collapsed ascending, transverse, and descending colon, along with ileal atresia type II (connected with a band) (Figure 4). An attempt to establish patency to the ascending and transverse colon was unsuccessful necessitating the placement of a cecostomy (Figure 5).

**Follow-up and outcome of interventions:** the patient resumed oral sips after fully awake from anesthesia, however, 32 hours post cecostomy she developed fever (temperature of 38.2°C), and diagnosis of septicemia was added and was treated. She was prescribed total parenteral nutrition (TPN) but she could not afford to buy it. Dressing using normal saline was done; and the patient resumed the course of antibiotics postoperative blood work-up control results were CBC (had normal leukocyte count 11, hemoglobin 19.5, platelets 370) electrolytes (sodium 130, potassium 5.4 and chloride 96) blood for culture and sensitivity result show no bacterial growth. The child kept deteriorating and passed away on day 3 post the cecostomy.

**Patient perspective:** despite the loss of their baby, the parents expressed gratitude for the efforts made by the team.

**Informed consent:** the parents provided informed consent for the publication.

## Discussion

A neonatal ileal atresia is a form of intestinal obstruction characterized by a blockage in the ileum, which can result from narrowing, complete closure, or absence of a segment of the ileum [3]. The incidence of intestinal atresia varies from 1 in 5000 to 1 in 14000 live births, with a higher prevalence in males compared to females, with a male-to-female ratio of 2:1 [4]. This condition can occur in any segment of the small bowel, either as a singular lesion or multiple lesions. Distal atresia typically presents delayed symptoms compared to proximal atresia. In certain instances, jejunoileal atresia may be associated with other congenital abnormalities such as cardiac anomalies, gastroschisis, and cystic fibrosis [5]. There are five main classifications of jejunoileal atresia: type I involves a membrane that fully obstructs the intestinal lumen while maintaining the integrity of the intestine. Type II is characterized by a discontinuity in the intestine with a fibrous cord connecting the proximal and distal segments, as observed intraoperatively in this case (Figure 4). Type IIIA is characterized by a mesenteric gap without any linkage between the segments. Type IIIB presents jejunal atresia with the absence of the distal superior mesenteric artery, resulting in the distal small bowel coiling like an apple peel and a shortened gut. Type IV comprises multiple atretic segments, resembling a string of sausages [2].

The diagnosis of neonatal ileal atresia relies on clinical history, physical examination, and imaging studies like abdominal X-rays and ultrasound. Clinical manifestations may include bilious vomiting, abdominal distension, and failure to pass meconium, as observed in this case report [6]. In a significant number of cases (29-50% involving jejunoileal atresia), prenatal detection is possible through fetal ultrasound, revealing signs such as polyhydramnios, ascites, dilated bowel loops, and increased bowel echogenicity [7]. Regrettably, in this case report, the mother did not undergo an obstetric ultrasound examination antenatally.

Supportive treatment for neonatal ileal atresia includes intravenous fluids, nasogastric decompression, and antibiotics. Definitive treatment involves a surgical intervention to remove the obstruction and restore normal intestinal continuity this may be achieved either with primary anastomosis or stoma creation first [8,9]. A decision to do a cecostomy was made in this case (Figure 5) due to the location of the lesion and the significant size discrepancy between the proximal and distal bowel.

The mortality rate after surgery for neonatal ileal atresia is relatively low, with most studies reporting a mortality rate of less than 5% attributed to advancements in neonatal intensive care, anesthesia, surgical techniques, and the utilization of total parenteral nutrition (TPN) [5]. However, the outcome may vary depending on the presence of associated anomalies and the overall health of the infant; our facility faced challenges in delivering optimal supportive care due to financial limitations and the unavailability of TPN. As a result, we had to rely on a nil per-oral protocol and intravenous fluids, which posed challenges in providing adequate nutritional support and correcting electrolyte imbalances post-surgery.

## Conclusion

Neonatal ileal atresia typically has a favorable prognosis when diagnosed early and promptly treated, thus promoting and encouraging the involvement of women in prenatal screening, specifically through fetal assessment in antenatal obstetrics ultrasound, can improve the detection of congenital conditions such as ileal atresia, leading to better outcomes. Nevertheless, in resource-constrained settings with limited access to total parenteral nutrition, ileal atresia continues to pose a significant challenge with a less favorable prognosis.
